# Impact of Age and Alberta Stroke Program Early Computed Tomography Score 0 to 5 on Mechanical Thrombectomy Outcomes: Analysis From the STRATIS Registry

**DOI:** 10.1161/STROKEAHA.120.032430

**Published:** 2021-06-03

**Authors:** Osama O. Zaidat, David S. Liebeskind, Ashutosh P. Jadhav, Santiago Ortega-Gutierrez, Thanh N. Nguyen, Diogo C. Haussen, Dileep R. Yavagal, Michael T. Froehler, Reza Jahan, Raul G. Nogueira, Tom L. Yao, Bader A. Alenzi, Saif Bushnaq, Nils H. Mueller-Kronast

**Affiliations:** 1 Neuroscience Institute, St Vincent Mercy Hospital, Toledo, OH (O.O.Z., S.B.).; 2 Neurovascular Imaging Research Core and Stroke Center, Department of Neurology (D.S.L.), University of California Los Angeles.; 3 Department of Radiology (R.J.), University of California Los Angeles.; 4 Department of Neurology, University of Pittsburgh Medical Center, PA (A.P.J.).; 5 Departments of Neurology, Neurosurgery and Radiology, University of Iowa Health Care, Carver College of Medicine (S.O.-G.).; 6 Division of Interventional Neuroradiology and Interventional Neurology, Boston Medical Center, MA (T.N.N.).; 7 Department of Neurology, Emory University, Atlanta, GA (D.C.H., R.G.N.).; 8 Department of Neurology and Neurosurgery, University of Miami Miller School of Medicine, FL (D.R.Y.).; 9 Cerebrovascular Program, Vanderbilt University Medical Center, Nashville, TN (M.T.F.).; 10 Norton Neuroscience Institute, Norton Healthcare, Louisville, KY (T.L.Y.).; 11 Department of Internal Medicine, The Ohio State University Wexner Medical Center, Columbus (B.A.A.).; 12 Advanced Neuroscience Network/Tenet South Florida, Boynton Beach (N.H.M.-K.).

**Keywords:** age, cerebral infarction, intracranial hemorrhage, ischemic stroke, large core infarction, thrombectomy

## Abstract

**Background and Purpose:**

This study investigates clinical outcomes after mechanical thrombectomy in adult patients with baseline Alberta Stroke Program Early CT Score (ASPECTS) of 0 to 5.

**Methods:**

We included data from the STRATIS Registry (Systematic Evaluation of Patients Treated With Neurothrombectomy Devices for Acute Ischemic Stroke) from patients who underwent mechanical thrombectomy within 8 hours of symptom onset and had available ASPECTS data adjudicated by an independent core laboratory. Angiographic and clinical outcomes were collected, including successful reperfusion (modified Thrombolysis in Cerebral Infarction ≥2b), functional independence (modified Rankin Scale score 0–2), 90-day mortality, and symptomatic intracranial hemorrhage at 24 hours. Outcomes were stratified by ASPECTS scores and age.

**Results:**

Of the 984 patients enrolled, 763 had available ASPECTS data. Of these patients, 57 had ASPECTS of 0 to 5 with a median age of 63 years (interquartile range, 28–100), whereas 706 patients had ASPECTS of 6 to 10 with a median age of 70 years of age (interquartile range, 19–100). Ten patients had ASPECTS of 0 to 3 and 47 patients had ASPECTS of 4 to 5 at baseline. Successful reperfusion was achieved in 85.5% (47/55) in the ASPECTS of 0 to 5 group. Functional independence was achieved in 28.8% (15/52) in the ASPECTS of 0 to 5 versus 59.7% (388/650) in the 6 to 10 group (*P*<0.001). Mortality rates were 30.8% (16/52) in the ASPECTS of 0 to 5 and 13.4% (87/650) in the 6 to 10 group (*P*<0.001). sICH rates were 7.0% (4/57) in the ASPECTS of 0 to 5 and 0.9% (6/682) in the 6 to 10 group (*P*<0.001). No patients aged >75 years with ASPECTS of 0 to 5 (0/12) achieved functional independence versus 44.8% (13/29) of those age ≤65 (*P*=0.005).

**Conclusions:**

Patients <65 years of age with large core infarction (ASPECTS 0–5) have better rates of functional independence and lower rates of mortality compared with patients >75 years of age.

**Registration:**

URL: https://www.clinicaltrials.gov; Unique identifier: NCT02239640.

## Abstract 2

Supplemental Digital Content is available in the text.

**See related article, p 2229**

Endovascular therapy for acute ischemic stroke (AIS) is the standard of care in adult patients with Alberta Stroke Program Early CT Score (ASPECTS) of 6 to 10 and large vessel occlusion, based on American Heart Association/American Stroke Association guidelines.^1,2^ These guidelines are based on several randomized clinical trials and endovascular therapy trials demonstrating superior efficacy of mechanical thrombectomy (MT) compared with medical therapy.^3–10^ However, the presenting core infarction volume beyond which MT is futile or potentially harmful has not been established.^11–13^ Data supporting MT use on low ASPECTS of 0 to 5 and its associated clinical outcomes is not conclusive, possibly due to the low volume of salvageable brain tissue and large core infarct volume, both of which predict a low functional independence rate and higher symptomatic intracranial hemorrhage (sICH) rate.^11–13^

In this study, we evaluated clinical outcomes in patients with AIS undergoing MT and presenting with large core infarct volume, as determined by a low ASPECTS (0–5), in the STRATIS Registry (Systematic Evaluation of Patients Treated With Neurothrombectomy Devices for Acute Ischemic Stroke).^14^ We aimed to assess whether low ASPECTS was associated with worse functional outcome and higher rates of sICH compared with patients with higher ASPECTS. Furthermore, because advanced age is associated with low rates of functional independence and high rates of sICH and mortality,^15–17^ we studied the combined effect of ASPECTS on the clinical outcome of MT stratified by age.

## Methods

### Data Availability

Requests for data access may be sent to the corresponding author.

### Study Design and Participants

The STRATIS registry was a prospective, single-arm, multicenter, nonrandomized, observational study that evaluated the use of Solitaire Revascularization Device (Solitaire, Medtronic, Minneapolis, MN) and Mindframe Capture Low profile Revascularization (Mindframe, Medtronic, Minneapolis, MN) in 1000 patients with anterior circulation emergent large vessel occlusion at 55 centers within the United States between August 2014 and June 2016. Ethic committees and institutional review boards approval was obtained at each medical center. Subjects were provided a written informed consent before enrollment. The details and results of the STRATIS Registry are published elsewhere.^14^

Patients who underwent MT per the study protocol were included based on the following inclusion criteria: (1) availability of ASPECTS before thrombectomy; (2) availability of angiographic data and clinical outcomes; (3) confirmed symptomatic intracranial emergent large vessel occlusion involving the internal carotid artery terminus or proximal middle cerebral artery; (4) confirmed National Institutes of Health Stroke Scale (NIHSS) scores of 8 to 30; (5) use of Medtronic market-released neurothrombectomy device as the initial device; (6) premorbid modified Rankin Scale (mRS) score of ≤1; and (7) arterial puncture within 8 hours of stroke onset.

### Imaging Analysis

Angiographic procedural images, baseline images (before MT), and follow-up (after MT) parenchymal noncontrast computed tomography (NCCT) images were evaluated by an independent core laboratory blinded to clinical outcomes data. NCCT was evaluated, interpreted, and an Imaging Report Form was completed by an experienced physician (D.S. Liebeskind) using the ASPECTS scoring system. The reader evaluated each of the ten ASPECTS regions using all imaged slices.^18^ After adjusting the window and level setting to optimize ASPECTS grading, a region was scored negatively if an early ischemic change with clear hypoattenuation or loss of gray-white matter differentiation was identified. Areas with early ischemic signs were deducted from the total ASPECTS of 10 to obtain the final ASPECTS score.

Based on the core lab adjudicated baseline NCCT ASPECTS, patients were grouped using traditional dichotomization (ASPECTS 0–5 versus 6–10 groups), as well as a trichotomized scheme (ASPECTS 0–3 [very large stroke], 4–5 [moderate/large core infarct], and 6–10 [small/moderate core infarct] groups). The ASPECTS of 0 to 5 group was further stratified based on patient age at presentation in 3 groups (≤65 versus >65–75 versus >75).

### Study Outcomes

The primary outcome was functional independence (mRS score 0–2) at 90 days. Angiographic outcomes included (1) successful reperfusion (modified Thrombolysis in Cerebral Infarction ≥2b) and (2) complete reperfusion (modified Thrombolysis in Cerebral Infarction 3). Technical and procedural outcomes included (1) first-pass effect (achieving modified Thrombolysis in Cerebral Infarction ≥2b in the first pass),^19^ (2) number of passes, (3) use of rescue therapy, (4) embolization into new territory, and (5) time from puncture to reperfusion. Secondary clinical outcomes included mortality at 90 days. Safety outcomes included rate of sICH (hemorrhage with associated NIHSS score worsening of 4 or more points) based on 24-hour-NCCT post-MT.

### Statistical Analysis

Student *t* tests were used for normally distributed continuous variables, the Mann-Whitney test was used for ordinal or non-normal variables, and Fisher exact or χ^2^ tests were used for categorical variables. Multivariate analysis was performed via stepwise logistic regression. Initial variable selection included successful recanalization, sICH, IV tPA (intravenous tissue-type plasminogen activator), ASPECTS and those with *P*<0.1 from univariate analyses. All analyses were performed by an independent statistician using SAS software (SAS Institute, Cary, NC). *P*<0.05 were considered significant. All supporting data from this study are available within the article.

## Results

### Baseline Characteristics

**Figure 1.**
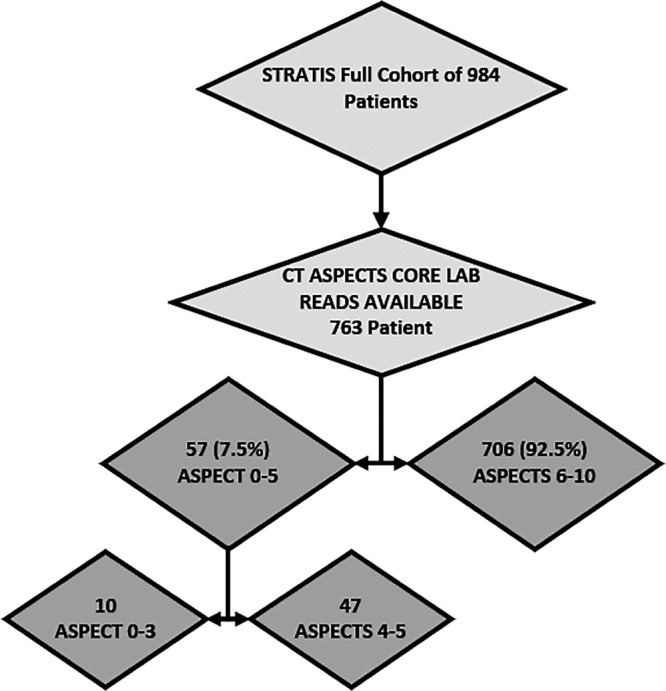
Low Alberta Stroke Program Early CT Score (ASPECTS) study flow chart.

Of the 984 patients enrolled in the STRATIS Registry, 763 (77.5%) had NCCT ASPECTS read by the imaging core lab and were included in this study (Figure 1). Of these patients, 57 (7.5%) had ASPECTS of 0 to 5 and 706 (92.5%) had ASPECTS of 6 to 10. Among the ASPECTS of 0 to 5 group, 10 (17.5%) patients had ASPECTS of 0 to 3 and 47 (82.5%) had ASPECTS of 4 to 5.

Baseline variables were compared among the ASPECTS of 0 to 5, and 6 to 10 groups (Table 1). Baseline variables further categorized into subgroups 0 to 3, 4 to 5, and 6 to 10 are found in Table I in the Data Supplement. Patients in the ASPECTS of 0 to 5 group were younger and had a more severe presentation versus the ASPECTS of 6 to 10 group (62.5±15.5 versus 68.5±14.9 years, *P*=0.009; mean baseline NIHSS 19.9±5.1 versus 17.0±5.4, *P*<0.001). Compared with the ASPECTS of 6 to 10 group, the ASPECTS of 0 to 5 cohort had significantly higher rates of internal carotid artery occlusions (22.0% [155/706] and 42.1% [24/57], *P*=0.002) and longer onset to puncture time (216.4±100.0 and 276.3±102.9 minutes, *P*<0.001).

**Table 1.**
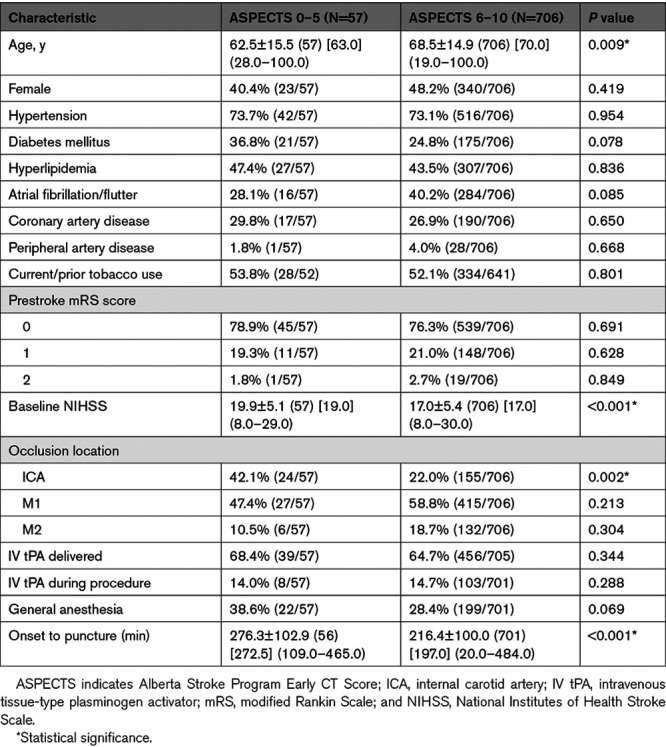
Baseline Variables Differences Between Patients by ASPECTS

### Angiographic and Technical Outcomes

Procedural, angiographic, and clinical outcome comparisons are presented in Table 2. There was no difference in successful reperfusion rate among these groups. Complete reperfusion rates were lower in the ASPECTS of 0 to 5 versus the ASPECTS of 6 to 10 group (0% [0/55] versus 12.5% [83/662], *P*=0.020). There was no difference in mean number of passes between the ASPECTS of 0 to 5 and ASPECTS of 6 to 10 group (2.0±1.4 versus 1.8±1.2, *P*=0.170). In a comparison of ASPECTS of 0 to 3, 4 to 5, and 6 to 10, there was no difference in first-pass effect rates between the ASPECTS of 0 to 3 and ASPECTS of 6 to 10 group (30% [3/10] versus 61% [425/697], *P*=0.056, Table II in the Data Supplement). There was a significantly higher rate of vessel cutoff downstream in the ASPECTS of 4 to 5 versus ASPECTS of 6 to 10 (73.3% [33/45] versus 56.6% [375/662], *P*=0.029).

**Table 2.**
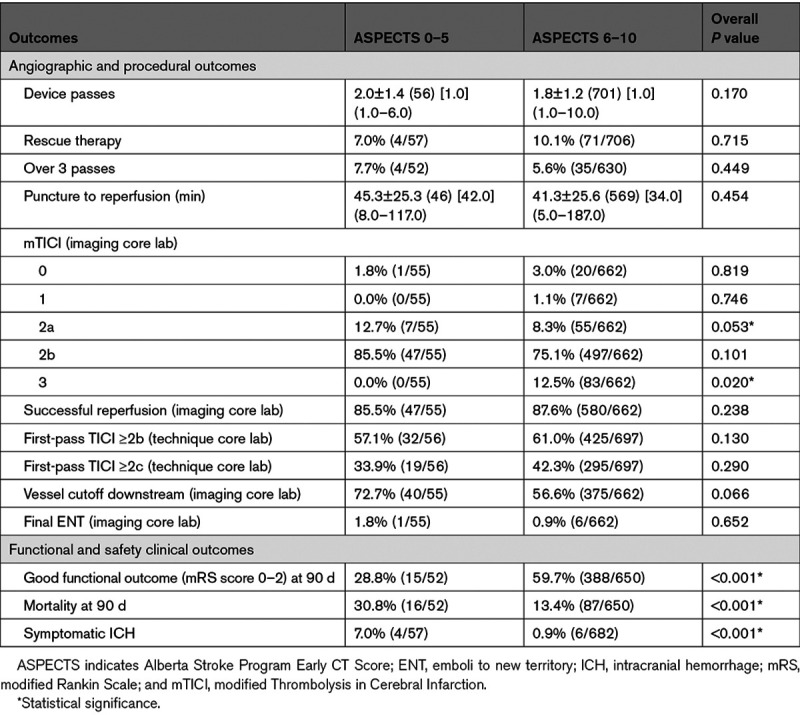
Angiographic, Procedural, and Clinical Outcomes in Patients Stratified by ASPECTS

### Functional and Safety Outcomes

**Figure 2.**
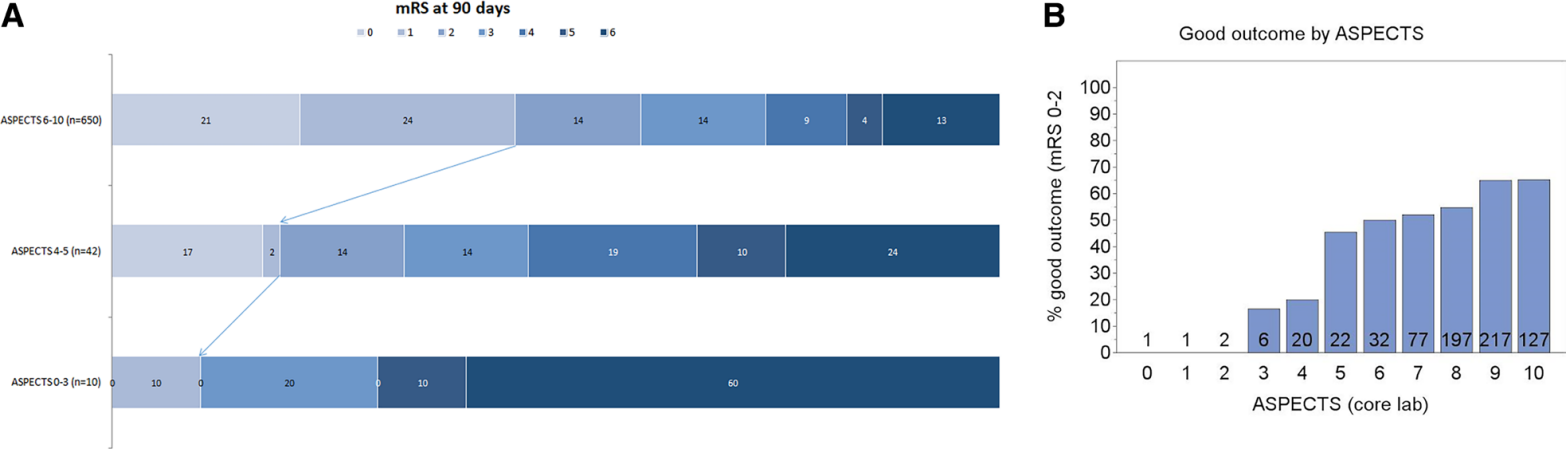
**Functional outcome (modified Rankin Scale [mRS]) at 90 d in patients with Alberta Stroke Program Early CT Score (ASPECTS) of 6–10, 4–5, and 0–3 (A).** Functional independence (mRS score 0-2) at 90 d by ASPECTS score. Number of patients corresponding to each ASPECTS is listed above the baseline (**B**).

Clinical and safety outcome comparisons are presented in Table 2. Among 57 patients with ASPECTS of 0 to 5, 90-day outcome was reported in 52 (91.2%) patients. There was a significantly lower rate functional independence in the ASPECTS of 0 to 5 cohort versus the ASPECTS of 6 to 10 group (28.8% versus 59.7%; *P*<0.001). There was no difference in functional independence rates between the ASPECTS of 0 to 3 group and the ASPECTS of 4 to 5 group (10.0% [1/10] and 33.4% [14/42]; *P*=0.247, Table II in the Data Supplement). An analysis of functional outcome at 90 days stratified by ASPECTS is summarized in Figure 2. Mortality at 90 days was significantly higher in the ASPECTS of 0 to 5 group (30.8%, 16/52) compared with the ASPECTS of 6 to 10 group (13.4%, 87/650; *P*<0.001). Mortality at 90 days was significantly higher in the ASPECTS of 0 to 3 group (60.0%, 6/10) compared with the ASPECTS of 6 to 10 group (13.4%, 87/650; *P*=0.001), although the small sample size of the ASPECTS of 0 to 3 group should be noted (Table II in the Data Supplement). sICH rate was 7.0% (4/57) in ASPECTS of 0 to 5 versus 0.9% (6/682) in the ASPECTS of 6 to 10 (*P*<0.001; Table 2).

### Procedural and Clinical Outcomes

Procedural, clinical, and safety outcome comparisons are presented in Table 3. There was no difference in baseline characteristics among different age strata within the ASPECTS of 0 to 5 group. However, none of the patients with ASPECTS of 0 to 5 and aged >75 years (0/12) had functional independence at 90 days compared with 44.8% (13/29) of those aged ≤65 (*P*=0.005) and 18.2% (2/11) of those aged >65 to 75 years (*P*=0.122) achieved functional independence. Within the ASPECTS of 0 to 5 group, patients >75 years had higher rates of mortality versus patients >65 to 75 and ≤65 years of age (7/12 [58.3%], 3/11 [27.3%], and 6/29 [20.7%], respectively).

**Table 3.**
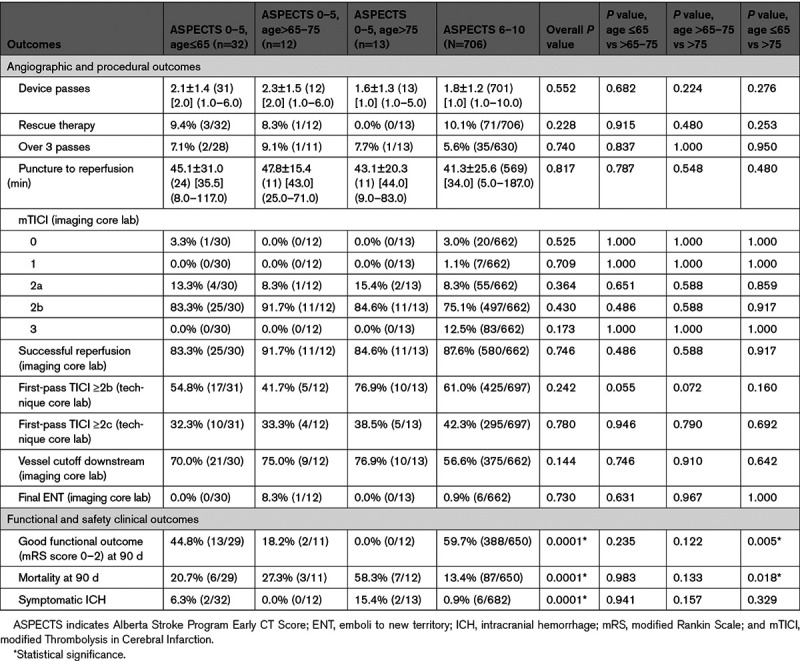
Angiographic, Procedural, and Clinical Outcomes in Patients With Low ASPECTS Stratified by Age

### Multivariate Analysis

ASPECT 0 to 3 (*P*=0.039) and 4 to 5 (*P*=0.007) were independent predictors of poor functional outcome (mRS score >2) at 3 months after adjusting for age, baseline NIHSS, successful reperfusion, sICH, onset to groin puncture, IV tPA administration, and general anesthesia (Table 4).

**Table 4.**
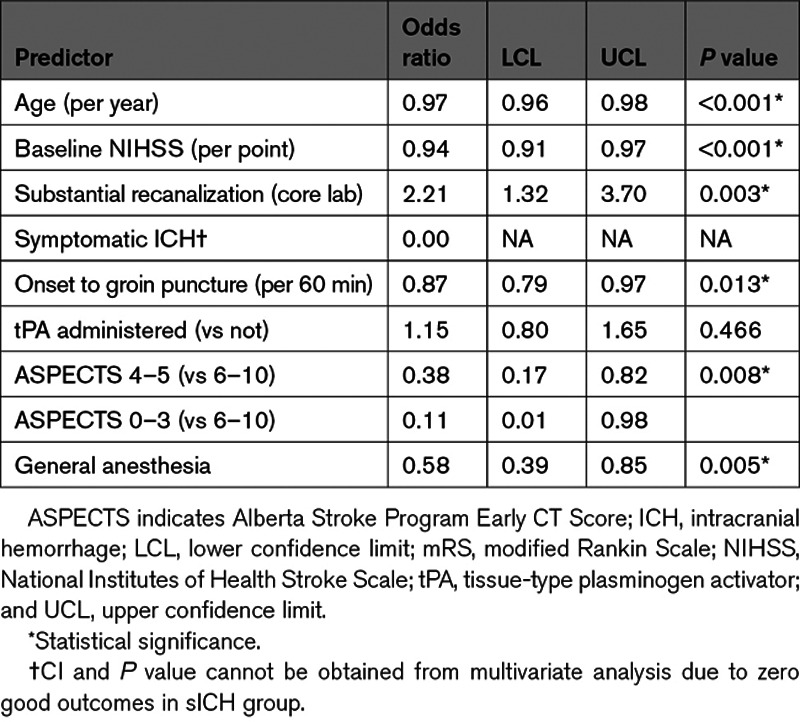
Multivariate Analysis Demonstrating Significant Effect of Low ASPECTS on Predicting Good Clinical Outcome (mRS Score 0–2)

## Discussion

Our study showed that ASPECTS of 0 to 5 is associated with a low good functional outcome rate and high mortality rate in patients >75 years, indicating a lower clinical benefit of MT in this patient population. Conversely, patients ≤65 years of age with ASPECTS of 0 to 5, had similar rates of both good functional outcome and mortality relative to the overall patient population and were significantly better than patients >75 years. Younger age, low baseline NIHSS score, absence of symptomatic ICH, and early time from onset to groin puncture were each shown to be important and independent predictors of good functional outcome; accordingly, regardless of ASPECTS score, patients demonstrating these characteristics are likely to have favorable prognosis.

ASPECTS as a surrogate marker of core infarct volume correlates with clinical outcomes in patients with AIS.^18^ However, the exact ASPECT score beyond which there is no clinical benefit from MT is not well-established since most recent clinical trials excluded patients with low ASPECTS (0–5).^1,3–11,13^ Limited data with small sample sizes preclude our ability to address this question that has both clinical and health care cost-related consequences.^20^ This is also a high-priority research question according to the Stroke Therapy Academic Industry Roundtable group.^21^

The low good functional outcome rate in patients with low ASPECTS is consistent with other studies. In a meta-analysis of 13 articles correlating baseline ASPECTS with clinical outcomes, mRS score of 0 to 2 was achieved in 17.1%, 35.7%, and 49.7% in the low (0–4), intermediate (5–7), and high (8–10) ASPECTS groups, respectively.^22^ Another meta-analysis of 17 studies reported mRS score of 0 to 2 for 1378 patients with ASPECTS of 0 to 6; mRS score of 0 to 2 rate was 37.7% for ASPECTS of 6, 33.3% for ASPECTS of 5, 22.1% for ASPECTS of 4, 17.1% for ASPECTS of 0 to 4, and 13.9% for ASPECTS of 0 to 3 groups.^23^ These findings, in combination with the present study, suggest patients with low baseline ASPECTS tend to have worse outcomes after MT. A subgroup analysis of a large multicenter retrospective registry (the Bernese-European Registry for Ischemic Stroke Patients Treated Outside Current Guidelines With Neurothrombectomy Devices using the SOLITAIRE FR With the Intention For Thrombectomy [BEYOND-SWIFT]) reported an overall mRS score of 0 to 2 in 24.6% patients with ASPECTS of 0 to 5 at 90 days, with a higher likelihood of favorable outcome (mRS score 0–3) in reperfused patients (Thrombolysis in Cerebral Infarction 2b–3) than nonreperfused (35.2% versus 8.3%, *P*=0.014), and comparable to those with ASPECTS of 6 to 10 (*P*=0.441).^11^ Moreover, reperfusion in patients with ASPECTS of 0 to 4 was not associated with good functional outcome (adjusted odds ratio [OR], 3.971 [95% CI, 0.951–16.585]), but mortality rate was significantly better than those without reperfusion (adjusted OR, 0.168 [95% CI, 0.056–0.499]).^11^ However, this study determined ASPECTS based on NCCT and had a relatively limited sample of patients with low ASPECTS.

The HERMES (Highly Effective Reperfusion Using Multiple Endovascular Devices) patient-level meta-analysis included both diffusion-weighted imaging- and CT-ASPECTS and reported a potential threshold of benefit (mRS shift) for ASPECTS of 3 to 5 (31% in MT versus 16% in IV tPA group (common OR, 2.00 [CI, 1.16–3.46]).^24^ However, there was no remarkable benefit of MT for patients with ASPECTS of 0 to 2 (0% in MT versus 12% in the control group (adjusted OR, 0.00 [CI, 0.00–5.81]).^24^ In the MR-CLEAN trial (Multicenter Randomized Clinical Trial of Endovascular Treatment for Acute Ischemic Stroke in the Netherlands), 6% (30/496) patients with ASPECTS of 0 to 4 were enrolled; 11 (36.7%) in the MT and 19 (6.3%) in the IV tPA treatment group, and mRS score of 0 to 2 was achieved in 1/11 (9%) of patients in the MT group versus 0/19 (0%) in the control group at 90 days.^13^ These data suggest marginal or potentially no benefit of MT in patients with ASPECTS of 0 to 2 or 0 to 3. However, nonrandomized studies and meta-analyses evaluating the clinical benefit of MT in patients with low ASPECTS have mixed results, with some studies demonstrating improvement after MT even in patients with incomplete or failed recanalization, while others suggest MT is not beneficial in patients with low ASPECTS.^13,22–25^

We showed that mortality rate increased with decreasing baseline ASPECTS; however, there was a significant increase in sICH rate in both the ASPECTS of 0 to 6 versus 6 to 10 and ASPECTS of 4 to 5 versus 6 to 10 groups. However, the HERMES analysis of ASPECTS of 0 to 4 patients showed a higher sICH rate in the MT group (19.2% versus 5.0%, respectively; adjusted common OR, 3.94 [95% CI, 0.94–16.49], *P*_interaction_=0.025), but no difference in mortality between MT versus control group (31.6%, and 35.7%, respectively; OR, 0.81 [95% CI, 0.36–1.81]; *P*=0.509).^24^ However, in the MR-CLEAN trial, the ASPECTS of 0 to 4 cohort did not show a significant difference in rates of sICH or 90-day mortality (4/11 [36%] in the MT group versus 8/19 [42%] in the IV tPA group).^13^

Although advanced age is associated with worse outcome, patients with advanced age still benefit from MT compared with those treated with IV tPA alone.^15,24^ Conversely, in the present study, functional independence rate decreased with increasing age in patients with low ASPECTS. This is consistent with the reduced good outcome (mRS score 0–2) rate in patients aged >70 years versus those aged <70 years (16.2% versus 40.3%, respectively), as reported in a recent meta-analysis by Cagnazzo et al.^23^ Additionally, in the low ASPECTS (0–5) subgroup analysis of the RESCUE-Japan (Recovery by Endovascular Salvage for Cerebral Ultra-Acute Embolism) Registry 2, patients aged <75 years were more likely to have favorable outcome with MT (OR, 2.43 [CI, 0.98–6.01]) versus those aged ≥75 years (OR, 2.11 [CI, 0.51–8.78]).^26^ Danière et al^27^ conducted a low diffusion-weighted imaging-ASPECT (<5) study, reporting that only 10% (12/120) of patients >70 years achieved good outcome (mRS score 0–2). In an analysis of the MR-CLEAN registry, the authors found that there was no significant interaction between age and ASPECTS on mRS outcomes, indicating that endovascular therapy should not be withheld from elderly patients with low ASPECTS.^28^ However, the authors did report an increased chance of sICH in elderly patients (age >71.8 years) with low ASPECTS.^28^

One limitation of our study is that patients from the STRATIS Registry with NCCT ASPECTS that were read by an independent core lab (763/984 [77.5%]) only, yielding low ASPECTS treated rate with MT of 7.5%. However, this number is consistent with low ASPECTS patient enrollment rate of 6% and 10% in the MR-CLEAN trial and HERMES collaborative meta-analysis, respectively.^13,24^ Moreover, the stratification of ASPECTS of 0–3 treated with MT only contained ten patients, making it difficult to draw conclusions from such a small sample size. Because our study uses registry-based data with less stringent criteria than randomized controlled trials, there are inherent selection biases. Additionally, we restricted the ASPECTS analysis on NCCT versus using diffusion-weighted imaging-ASPECTS, which may have yielded different efficacy and safety results of MT.^27^ Our analysis also did not analyze infarcts by topographical region. The analysis of the NCCT by a single physician is a limitation, due to variation in ASPECTS analysis between observers.^29^ Future research may include artificial intelligence semi-quantification of ASPECT score using unapproved tools that are currently available for research only such as RAPID-ASPECTS, e-ASPECTS, or VIZ-AI-ASPECTS. Another limitation is that low ASPECTS may not be as predictive of ischemic core volume, which has been reported with diffusion-weighted imaging-ASPECTS.^29^

## Conclusions

Our study demonstrated poor MT outcomes for AIS patients with low baseline ASPECTS (0–5), with poor functional independence rate and high rates of mortality and sICH. Moreover, patients with ASPECTS of 0 to 3 and patients >75 years of age had worse rates of functional independence and mortality among patients with ASPECTS of 0 to 5. Prospective randomized studies are warranted to establish the impact of low ASPECTS on MT outcomes in patients with AIS.

## Acknowledgments

We acknowledge Oscar H. Bolanos, Medtronic, and Meliza Ward (St Vincent Hospital) for editorial support.

## Sources of Funding

This study was sponsored by Medtronic, Inc.

## Disclosures

Dr Zaidat serves as a consultant for Neuravi, Stryker, Penumbra, and Medtronic. Dr Zaidat received personal fees from Medtronic during the conduct of the study and also received research grants from the TESLA (Thrombectomy for Emergent Salvage of Large Anterior Circulation Ischemic Stroke). Dr Liebeskind serves as a consultant for Cerenovus, Genentech, Medtronic, Stryker, and Vesalio. Dr Ortega-Gutiérrez serves as a consultant for Medtronic and Stryker Neurovascular and has received personal fees from both. Dr Nguyen is the Principal Investigator of the CLEAR study (CT for Late Endovascular Reperfusion) funded by Medtronic; serves on the Data Safety Monitoring Board for TESLA, ENDOLOW (Endovascular Therapy for Low NIHSS Ischemic Strokes), SELECT 2 (A Randomized Controlled Trial to Optimize Patient’s Selection for Endovascular Treatment in Acute Ischemic Stroke) trials. Dr Haussen serves as a consultant for Stryker, Vesalio, and Cerenovus; has stock options with Viz-Ai. Dr Yavagal serves as a consultant for Medtronic, Cerenovus, Rapid Medical, Vascular Dynamics, scientific advisory board member for Poseydon, Neurosave, and Neuralanalytics. Dr Froehler serves as a scientific consultant to Medtronic, Stryker, Balt USA, Viz.ai, Corindus, and Genentech and has received research funding from the National Institutes of Health (NIH). Dr Jahan serves as a consultant for Stryker, Medtronic, Microvention, and Genentech. Dr Jahan receives funding for RJ’s services as a scientific consultant regarding trial design and conduct to Medtronic/Covidien; he is an employee of the University of California, which holds a patent on retriever devices for stroke. Dr Nogueira has served as an advisor or consultant for Cerenovus/Neuravi, Phenox, Stryker Neurovascular and owns stock, stock options, or bonds from Cerenovus/Neuravi, Phenox, and Stryker Neurovascular. Dr Nogueira also has received consulting fees for advisory roles with Anaconda, Biogen, Genentech, Imperative Care, Medtronic, Prolong Pharmaceuticals, and stock options for advisory roles with Astrocyte, Brainomix, Cerebrotech, Ceretrieve, Corindus Vascular Robotics, Vesalio, Viz-AI, and Perfuze. Dr Yao serves as consultant/proctor for Medtronic and has received fees from Medtronic and Microvention. Dr Mueller-Kronast serves as scientific consultants regarding trial design and conduct to Medtronic. The other authors report no conflicts.

## Supplemental Materials

Online Tables I–II

## Supplementary Material


