# Supplementary material for: Impact of Age and Alberta Stroke Program Early Computed Tomography Score 0 to 5 on Mechanical Thrombectomy Outcomes: Analysis From the STRATIS Registry
Source: Stroke. 2021 Jun 3;52(7):2220–8. doi: 10.1161/STROKEAHA.120.032430 (PMC8240495; doi:10.1161/STROKEAHA.120.032430)
Supplement: Supplementary file 1 [file str-52-2220-s001.pdf]

SUPPLEMENTAL MATERIAL

Impact of Age and ASPECTS 0-5 on Mechanical Thrombectomy Outcomes: Analysis from the STRATIS Registry

Contents:

Table I Baseline characteristics stratified by ASPECTS

Table II Angiographic, Procedural, and Clinical Outcomes in Patients Stratified by ASPECTS

Table I Baseline characteristics stratified by ASPECTS

Characteristic	ASPECTS 0-3 (n=10)	ASPECTS 4-5 (n=47)	ASPECTS 6-10 (N=706)	p-value 0-3 vs 4-5	p-value 0-3 vs 6-10	p-value 4-5 vs 6-10
**Age (years)**	64.4±18.2 (10) [72.0] (28.0,83.0)	62.1±15.1 (47) [62.0] (29.0,100.0)	68.5±14.9 (706) [70.0] (19.0,100.0)	0.675	0.385	0.004
Female	50.0% (5/10)	38.3% (18/47)	48.2% (340/706)	0.504	1.000	0.228
Hypertension	70.0% (7/10)	74.5% (35/47)	73.1% (516/706)	0.713	0.734	1.000
Diabetes mellitus	50.0% (5/10)	34.0% (16/47)	24.8% (175/706)	0.473	0.132	0.167
Hyperlipidemia	50.0% (5/10)	46.8% (22/47)	43.5% (307/706)	1.000	0.754	0.653
Atrial fibrillation/flutter	10.0% (1/10)	31.9% (15/47)	40.2% (284/706)	0.253	0.058	0.285
Coronary artery disease	40.0% (4/10)	27.7% (13/47)	26.9% (190/706)	0.464	0.472	0.867
Peripheral artery disease	0.0% (0/10)	2.1% (1/47)	4.0% (28/706)	1.000	1.000	1.000
Current/prior tobacco use	44.4% (4/9)	55.8% (24/43)	52.1% (334/641)	0.716	0.744	0.753
Pre-stroke mRS						
0	70.0% (7/10)	80.9% (38/47)	76.3% (539/706)	0.424	0.708	0.594
1	30.0% (3/10)	17.0% (8/47)	21.0% (148/706)	0.387	0.447	0.583
2	0.0% (0/10)	2.1% (1/47)	2.7% (19/706)	1.000	1.000	1.000
Baseline NIHSS	22.0±4.2 (10) [21.5] (16.0,28.0)	19.4±5.2 (47) [19.0] (8.0,29.0)	17.0±5.4 (706) [17.0] (8.0,30.0)	0.149	0.004	0.003

Characteristic	ASPECTS 0-3 (n=10)	ASPECTS 4-5 (n=47)	ASPECTS 6-10 (N=706)	p-value 0-3 vs 4-5	p-value 0-3 vs 6-10	p-value 4-5 vs 6-10
Occlusion location						
ICA	50.0% (5/10)	40.4% (19/47)	22.0% (155/706)	0.727	0.050	0.007
M1	40.0% (4/10)	48.9% (23/47)	58.8% (415/706)	0.734	0.333	0.222
M2	10.0% (1/10)	10.6% (5/47)	18.7% (132/706)	1.000	0.698	0.239
IV-tPA delivered	50.0% (5/10)	72.3% (34/47)	64.7% (456/705)	0.260	0.338	0.344
IA-tPA during procedure	30.0% (3/10)	10.6% (5/47)	14.7% (103/701)	0.137	0.176	0.527
General anesthesia	60.0% (6/10)	34.0% (16/47)	28.4% (199/701)	0.161	0.038	0.408
Onset to puncture (min)	261.1±108.2 (9) [247.0] (145.0,452.0)	279.2±102.8 (47) [289.0] (109.0,465.0)	216.4±100.0 (701) [197.0] (20.0,484.0)	0.634	0.184	<0.001

ASPECTS, Alberta Stroke Program Early CT Score; mRS, modified Rankin Scale; NIHSS, National Institutes of Health Stroke Scale; ICA, Internal carotid artery; IV-tPA, Intravenous-tissue plasminogen activator.

Table II Angiographic, Procedural, and Clinical Outcomes in Patients Stratified by ASPECTS

**Outcomes**	**ASPECTS 0-3**	**ASPECTS 4-5**	**ASPECTS 6-10**	**p-value 0-3 vs 4-5**	**p-value 0-3 vs 6-10**	**p-value 4-5 vs 6-10**
Angiographic and Procedural Outcomes						
Device passes	2.2±1.0 (10) [2.0] (1.0,4.0)	2.0±1.5 (46) [1.0] (1.0,6.0)	1.8±1.2 (701) [1.0] (1.0,10.0)	0.686	0.317	0.326
Rescue therapy	10.0% (1/10)	6.4% (3/47)	10.1% (71/706)	0.548	1.000	0.612
Over 3 passes	0.0% (0/9)	9.3% (4/43)	5.6% (35/630)	1.000	1.000	0.304
Puncture to reperfusion (min)	38.9±21.0 (7) [38.0] (8.0,71.0)	46.4±26.0 (39) [43.0] (9.0,117.0)	41.3±25.6 (569) [34.0] (5.0,187.0)	0.472	0.805	0.225
mTICI (imaging core lab)						
0	0.0% (0/10)	2.2% (1/45)	3.0% (20/662)	1.000	1.000	1.000
1	0.0% (0/10)	0.0% (0/45)	1.1% (7/662)	1.000	1.000	1.000
2a	30.0% (3/10)	8.9% (4/45)	8.3% (55/662)	0.104	**0.047**	0.783
2b	70.0% (7/10)	88.9% (40/45)	75.1% (497/662)	0.149	0.717	**0.046**
3	0.0% (0/10)	0.0% (0/45)	12.5% (83/662)	1.000	0.621	**0.006**
Successful reperfusion (imaging core lab)	70.0% (7/10)	88.9% (40/45)	87.6% (580/662)	0.149	0.121	1.000
First pass TICI≥2b (technique core lab)	30.0% (3/10)	63.0% (29/46)	61.0% (425/697)	0.080	0.056	0.876
First pass TICI≥2c (technique core lab)	20.0% (2/10)	37.0% (17/46)	42.3% (295/697)	0.467	0.205	0.539

Vessel cutoff downstream (imaging core lab)	70.0% (7/10)	73.3% (33/45)	56.6% (375/662)	1.000	0.527	**0.029**
Final ENT (imaging core lab)	0.0% (0/10)	2.2% (1/45)	0.9% (6/662)	1.000	1.000	0.370
**Functional and Safety Clinical Outcomes**						
Good functional outcome (mRS 0-2) at 90 days	10.0% (1/10)	33.3% (14/42)	59.7% (388/650)	0.247	**0.002**	**0.001**
Mortality at 90 days	60.0% (6/10)	23.8% (10/42)	13.4% (87/650)	**0.052**	**0.001**	**0.067**
Symptomatic ICH	0.0% (0/10)	8.5% (4/47)	0.9% (6/682)	1.000	1.000	**0.002**

ASPECTS, Alberta Stroke Program Early CT Score; ENT, emboli to new territory; mTICI, modified thrombolysis in cerebral infarction; ICH, intracranial hemorrhage; mRS, modified Rankin Scale.
