# Supplementary material for: Impact of Age and Alberta Stroke Program Early Computed Tomography Score 0 to 5 on Mechanical Thrombectomy Outcomes: Analysis From the STRATIS Registry
Source: Stroke. 2021 Jun 3;52(7):2220–8. doi: 10.1161/STROKEAHA.120.032430 (PMC8240495; doi:10.1161/STROKEAHA.120.032430)
Supplement: Supplementary file 2 [file str-52-2220-s002.pdf]

American Heart Association | American Stroke Association

Acknowledgment Permission Form

Journal Stroke
Manuscript Number STROKE/2020/032430R1
First Author Osama Zaidat
Title of Work Impact of Age and ASPECTS 0-5 on Mechanical Thrombectomy Outcomes: Analysis from the STRATIS Registry

Authors must provide written permission/approval from all individuals mentioned by name in the Acknowledgments section of a submitted manuscript. By signing this form, any and all acknowledged persons therefore state that they have read and approved the mention of their names in the Acknowledgment section of the aforementioned paper.

	Name	Signature	Date
Name (1)	Oscar Bolanos	Oscar Bolanos	01 / 28 / 2021
Name (2)	Meliza Ward	Meliza Ward	01 / 28 / 2021
Name (3)			
Name (4)			
Name (5)			
Name (6)			
Name (7)			
Name (8)			
Name (9)			
Name (10)			
Name (11)			
Name (12)			
Name (13)			
Name (14)			
Name (15)			
Name (16)			
Name (17)			
Name (18)			
Name (19)			
Name (20)			

Doc ID: 1f30f9d487f9fdf97ba14a8a6870a7b60c8e79a3

**TITLE**	Stroke--Acknowledgement Permission Form
**FILE NAME**	Acknowledgment Pe...1529339228403.pdf
**DOCUMENT ID**	1f30f9d487f9fdf97ba14a8a6870a7b60c8e79a3
**AUDIT TRAIL DATE FORMAT**	MM / DD / YYYY
**STATUS**	● Completed

Document History

SENT	**01** / **28** / **2021**<br>14:30:52 UTC-5	Sent for signature to Oscar Bolanos (oscar.bolanos@medtronic.com) from meliza@galaxytherapeutics.com<br>IP: 98.28.48.25
VIEWED	**01** / **28** / **2021**<br>15:39:00 UTC-5	Viewed by Oscar Bolanos (oscar.bolanos@medtronic.com)<br>IP: 75.84.91.90
SIGNED	**01** / **28** / **2021**<br>15:39:45 UTC-5	Signed by Oscar Bolanos (oscar.bolanos@medtronic.com)<br>IP: 75.84.91.90
COMPLETED	**01** / **28** / **2021**<br>15:39:45 UTC-5	The document has been completed.
